# Plant-Made Bet v 1 for Molecular Diagnosis

**DOI:** 10.3389/fpls.2019.01273

**Published:** 2019-10-10

**Authors:** Mattia Santoni, Maria Antonietta Ciardiello, Roberta Zampieri, Mario Pezzotti, Ivana Giangrieco, Chiara Rafaiani, Michela Ciancamerla, Adriano Mari, Linda Avesani

**Affiliations:** ^1^Department of Biotechnology, University of Verona, Verona, Italy; ^2^Institute of Bioscience and BioResources, CNR, Naples, Italy; ^3^ADL (Allergy Data Laboratories) S.r.l., Latina, Italy; ^4^Associated Centre for Molecular Allergology, Rome, Italy

**Keywords:** allergen, molecular farming, transient expression, IgE, structure homology

## Abstract

Allergic disease diagnosis is currently experiencing a breakthrough due to the use of allergenic molecules in serum-based assays rather than allergen extracts in skin tests. The former methodology is considered a very innovative technology compared with the latter, since it is characterized by flexibility and adaptability to the patient’s clinical history and to microtechnology, allowing multiplex analysis. Molecular-based analysis requires pure allergens to detect IgE sensitization, and a major goal, to maintain the diagnosis cost-effective, is to limit their production costs. In addition, for the production of recombinant eukaryotic proteins similar to natural ones, plant-based protein production is preferred to bacterial-based systems due to its ability to perform most of the post-translational modifications of eukaryotic molecules. In this framework, Plant Molecular Farming (PMF) may be useful, being a production platform able to produce complex recombinant proteins in short time-frames at low cost. As a proof of concept, PMF has been exploited for the production of Bet v 1a, a major allergen associated with birch (*Betula verrucosa*) pollen allergy. Bet v 1a has been produced using two different transient expression systems in *Nicotiana benthamiana* plants, purified and used in a new generation multiplex allergy diagnosis system, the patient-Friendly Allergen nano-BEad Array (FABER). Plant-made Bet v 1a is immunoreactive, binding IgE and inhibiting IgE-binding to the *Escherichia coli* expressed allergen currently available in the FABER test, thus suggesting an overall similar though non-overlapping immune activity compared with the *E. coli* expressed form.

## Introduction

Allergic diseases, defined as abnormal responses of the human body when in contact with an allergen, have become a common health problem worldwide, with a rising incidence both in adults and children (Lau et al., 2018). It is rather difficult to obtain exact epidemiology data on this topic but it has been estimated that 25% of the general population suffer from these diseases (Sampson, 2004).

The primary diagnostic tool currently used for allergic disease diagnosis is skin prick testing (SPT), which evaluates the presence and degree of cutaneous reactivity against a surrogate marker of sensitization, composed of protein extracts from allergenic sources. However, this method is hampered by several problems. For instance, it cannot be used in patients who have extensive eczema, dermographism or urticaria or who are taking antihistamines and/or other medications. More importantly, it frequently fails to detect specific IgE because the composition of the surrogate markers can be very variable (Giangrieco et al., 2012). This high variability makes it impossible to have standardized extracts with a constant allergen composition, since this may be influenced by several factors, such as differences among plant cultivars, post-harvest treatments, specific phenological stages, and extraction protocols (Ciardiello et al., 2009; Pasquariello et al., 2012). Molecule-based diagnosis may overcome all these drawbacks, given the possibility of detecting the specific IgE against individual allergenic molecules (Giangrieco et al., 2012; Ciardiello et al., 2013).

Molecular-based diagnosis, furthermore, may be used to predict biopharmaceuticals with a potential use in therapy for tolerance induction to allergens in a drug-companion diagnostic strategy.

The allergens used for molecular diagnosis may be extracted from the original sources or may be produced recombinantly in different platforms. A plant-based platform, in this framework, represents both a cost-effective and speedy system for producing allergens and, in particular, for the expression of plant allergens. In fact, it provides an environment useful to obtain features, such as post-translational modifications and protein folding similar to those present in the molecules of the authentic source.

In this study, we have transiently transformed *Nicotiana benthamiana* plants for the production of one of the major allergens associated with birch pollen allergy, Bet v 1a (UniProtKB accession number P15494) (Radauer et al., 2008). Bet v 1a is a 17-kDa protein which shares epitopes with the major pollen allergens of trees belonging to the Fagales order and with some plant-derived foods (Niederberger et al., 1998). Bet v l represents a target for IgE antibodies of more than 95% of patients allergic to birch pollen, and almost 60% of them are exclusively sensitized to Bet v 1 (Jarolim et al., 1989).

Here we report the setup of a plant-based system for allergen production that was tested with the expression of recombinant Bet v 1a. For this purpose, two different transient systems in terms of yield and timeframe for protein upstream processing were used, and the results were compared. In addition, the features of the recombinant product were characterized. In particular, the folding of the plant-made Bet v 1a (pBet v 1a) was investigated by circular dichroism measurements, whereas the evaluation of its immunological reactivity (IgE binding) was analyzed with the FABER multiplex system by direct testing and experiments of IgE binding inhibition.

## Materials and Methods

### Vectors and Plant Transformation

The DNA sequence encoding the allergen Bet v 1a was designed with the following modifications: the codon usage was optimized for *N. benthamiana* and a poly-Histidine tag, a Flag-tag, and a linker (GPGP) were added at the N-terminus. The synthetic gene (Invitrogen GeneArt Gene Synthesis) was then inserted into the pENTR™/D-TOPO vector, following the manufacturer’s instructions, and sequenced to assess the absence of errors. The resulting vector was recombined by Gateway™ LR Clonase? II Enzyme mix (ThermoFisher) in the two destination vectors pK7WG2 (Karimi et al., 2002) and pG PVX GATEWAY(A) (Avesani et al., 2007).

The final result consisted of two vectors, pK7WG2.Betv1 and pGPVXGATEWAY(A).Betv1, that were inserted into the *Agrobacterium tumefaciens*, EHA105 and GV3101 strains, respectively, by electroporation.

### pBet v 1a Transient Expression in *N. Benthamiana*

*Nicotiana benthamiana* plants were grown from seeds and cultivated in a growth chamber at 25°C with a light/dark cycle of 16 h/8 h and a relative humidity of 20% to 40%.

A. tumefaciens cells, both EHA105 and GV3101, carrying pK7WG2.Betv1 and pGPVXGATEWAY(A).Betv1 were seeded into a lysogeny broth (LB) medium containing 50 µg/ml of rifampicin, 300 µg/ml of streptomycin, and 100 µg/ml of spectomycin for pK7WG2.Betv1 or 50 µg/ml of rifampicin, 50 µg/ml of kanamycin, and 5 µg/ml of tetracyclin for pGPVXGAT(A).Betv1. Empty vectors were used as negative controls. For syringe agroinfiltration, performed as described in Gecchele et al. (2015), overnight bacterial cultures were collected by centrifugation at 4500g, re-suspended in the infiltration buffer (10 mM MES pH 5.5, 10 mM MgSO4, and 100 µM acetosyringone) at an optical density of 0.8 at 600 nm. Following a 3-h incubation, the culture was used for the syringe infiltratation of 4- to 5-week-old *N. benthamiana* plants.

After the infiltration, the plants carrying the pK7WG2.Betv1 vector were sampled from the third day post-inoculation (dpi) to the 14th dpi; the plants infiltrated with the pG PVX GATEWAY(A).Betv1 were harvested after the symptom appearance between 10 to 14 dpi.

### pBet v 1a Detection

Total soluble proteins (TSP) were extracted from the leaves by grinding the tissue sample to a fine powder under liquid nitrogen. The powder was re-suspended in three volumes of extraction buffer (1× phosphate-buffered saline [PBS], 0.1% Tween-20) supplemented with cOmplete™ EDTA-free protease inhibitor (COEDTAF-RO).

The homogenate was centrifuged at 30,000g for 20 min at 4°C. The protein concentration was determined using the Bradford reagent (Sigma B6916).

The presence of pBet v 1a in the homogenate was detected by Western blot analysis. Briefly, equal quantities of TSP were loaded onto a 14% reducing SDS-PAGE. After the electrophoretic separation, the proteins were transferred onto a nitrocellulose membrane by electroblotting and incubated with anti-polyHisitidine and anti-FLAG® antibodies, diluted 1:5000 and 1:1000, respectively. The protein band recognized by the antibodies was detected using the ECL™ Select Western Blotting Detection Reagent (Amersham). In particular, the chemiluminescent signal was captured with the Chemidoc™ (BioRad).

### pBet v 1a Immobilized Metal Affinity Chromatography (IMAC) Purification

The purification of the allergen was carried out as described in Bortesi et al. (2009). Briefly, 10 to 30 g of leaf tissue were homogenized in four volumes of buffer (1× PBS, 10 mM ascorbic acid, 0.1% Tween-20, pH 6.0). To remove the insoluble material, the homogenate was centrifuged at 15,000g, at 4°C for 15 min. Next, the supernatant was removed, passed through a filter paper and adjusted to 500 mM NaCl, 5 mM imidazole, pH 8. After incubation with gentle shaking for 1 h in ice, a last centrifugation step at 4°C for 30 min at 30,000g produced a clear supernatant. To remove non-specifically bound material, the supernatant was loaded onto a disposable column packed with 1 ml Ni-NTA resin (QIAGEN), using a gravity flow. The column was washed with a PBS solution containing 500 mM NaCl, 0.1% Tween-20, 10 mM imidazole, pH 8. pBet v 1a was eluted with an increasing concentration of imidazole, ranging from 10 to 200 mM, using a gradient maker. The purity of the allergen preparation was analyzed by loading aliquots of the eluted fractions on reducing SDS-PAGE, followed by silver staining (Mortz et al., 2001). The pure fractions were pooled and dialyzed against 1× PBS.

### Size Exclusion Chromatography

The purified Bet v 1a was subjected to size exclusion chromatography using a fast protein liquid chromatography (FPLC) system, model AKTA pure 25L- Gold seal (GE Healthcare Europe GmbH, Milan, Italy). The purified protein was loaded on a gel filtration column Superdex 75 HR10/30 (Amersham Biosciences, Uppsala, Sweden), equilibrated, and eluted with 10 mM Tris-HCl, pH 7.5, 0.25 M NaCl. Fractions of 0.5 ml were collected, and the absorbance at 280 nm was recorded.

### RP-HPLC Chromatography

Aliquots of the protein fraction collected from the size exclusion chromatography were subjected to RP-HPLC separation. The protein was loaded on a Vydac (Deerfield, IL, USA) C8 column (4.6 × 250 mm), using a Beckman System Gold apparatus (Fullerton, CA, USA). Elution was performed by a multistep linear gradient of eluent B (0.08% TFA in acetonitrile) in eluent A (0.1% TFA) at a flow rate of 1 ml/min. The eluate was monitored at 220 and 280 nm.

### Estimation of the Pure Protein Concentration

The concentration of the pure protein obtained from size exclusion chromatography was estimated on the basis of the molar extinction coefficient, at 280 nm (11,920 M·cm^−1^), calculated for pBet v 1a (178 residues) using the ProtParam tool on the Exapsy server (www.expasy.org).

### Mass Spectrometry Experiments

The protein sample (5 µg) deriving from the RP-HPLC elution was dried with a centrifugal vacuum concentrator (Savant Speedvac Plus SC110A, Ramsey, Minnesota, USA) and solubilized in ammonium bicarbonate (AMBIC) 0.1 M. Next, a standard protocol of reduction, alkylation, and digestion with trypsin was applied. Briefly, the protein sample was reduced with 1 mM dithiothreithol in 100 mM AMBIC and alkylated with 5.5 mM iodoacetamide (IAA) in 10% acetonitrile and 10 mM AMBIC. The protein was then desalted using ZipTip C18 tips (Millipore, Billerica, MA) and incubated overnight at a trypsin/substrate ratio of 1:50 at 37°C for 20 h. The obtained peptides were separated using an Ultimate 3000 instrument (LC Packings, Sunnyvale, CA) 2D-nano-HPLC online interfaced with a QSTAR-Elite Hewlett Packard, Series 1100 HPLC with UV detector. The peptide sequence identification was obtained using the Mascot Server (Matrix Science, London, UK) on the website www.matrixscience.com.

### Circular Dichroism (CD) Experiments

CD spectra were recorded on a JASCO J-810 spectropolarimeter (Easton, MD) as already reported (Offermann et al., 2015). A quartz cell of 0.1-cm path length was used to record the spectra over the wavelength range of 260 to 195 nm with a bandwidth of 1.0 nm and a time constant of 8.0 s. For the CD experiments, the protein solution was diluted in the appropriate buffer and left for 2 h at 25°C before the acquisition of the spectra. The measurements were performed in PBS containing 0.1% Tween at 25°C. The protein concentration was 0.10 mg/ml. Each spectrum was baseline corrected for the contribution of the solvent.

For the thermostability experiments, purified pBet v 1a (0.1 mg/ml) was incubated for 5 min in PBS containing 0.1% Tween, at different temperatures. After each incubation, the CD spectrum was recorded as described above.

### Specific IgE Detection by the FABER Testing System

FABER (ADL S.r.l., Latina, Italy) is a multiplex *in vitro* serological test that allows the detection of IgE antibodies produced by allergic subjects, specifically recognizing the allergens spotted on the biochip (Alessandri et al., 2017; Tuppo et al., 2018). The FABER version used to perform the present study (FABER 244-122-122) bears 244 allergenic preparations, representing 122 purified molecular allergens and 122 multiple protein allergenic extracts. The data were obtained using 304 sera and a set of biochips containing also Bet v 1-like allergens, namely, Act c 11 from gold kiwi, Api g 1 from celery, Ara h 8 from peanut, Cor a 1 from hazel pollen, and Mal d 1 from apple, and including pBet v 1a, spotted for experimental purposes. Before the immobilization on the FABER biochip, pBet v 1a was coupled on nanobeads following the same procedure applied to all the other allergenic preparations. This multiplex diagnostic test allowed the detection of specific IgE to each of the allergenic preparations contained in the FABER biochip, including Bet v 1 from *Escherichia coli* and that from the plant, in a single run. To obtain information on shared epitopes on homologous proteins a modified single point highest inhibition achievable assay (SPHIAa) was used (Bernardi et al., 2011). IgE binding inhibition on Bet v 1-like allergens was achieved by co-incubating pBet v 1a with 5 sera from Bet v 1-allergic subjects, all having Bet v 1-specific IgE antibodies. For control purposes non-related IgE-positive results were used, and no inhibition was recorded (data not shown). The optimal pBet v 1a concentration for the inhibition experiments was found by preliminary experiments using a range of concentrations between 100 µg and 1.25 µg/ml in a buffer solution. All IgE detections, either for the inhibition assay or the direct measurements, were performed in a single replicate.

Based on the current regulation on spared serum samples from the diagnostic workup, considering the venous blood sampling as part of the routine clinical practice and the observational nature of the study carried out without any action on patients themselves, a formal approval by the ethical committee or signed informed consent was not required.

## Statistics

Statistical evaluation of protein yields and IgE distribution was made by applying the t-test for paired values and a *p* < 0.05 was considered statistically significant (Graphpad Prism 5.0; Graphpad Software Inc., San Diego, CA).

## Results

### pBet v 1a Expression

Two different transient expression systems for the expression of Bet v1a in *N. benthamiana* plants were compared.

#### pK7WG2 Expression

**Figure 1:**
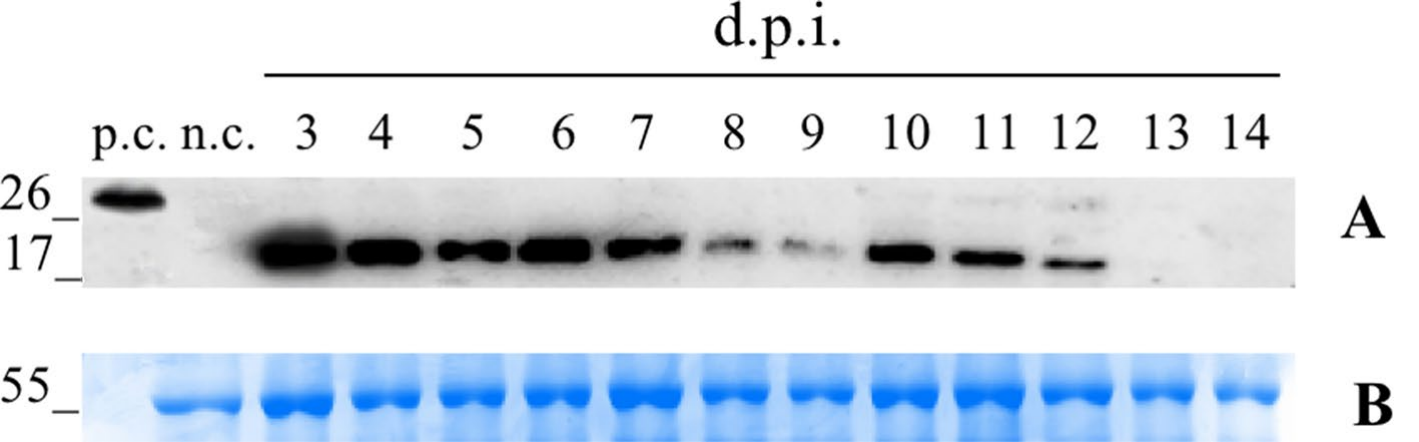
Time-course analysis of pK7WG2. Betv1 expression of the agro-infiltrated *N. benthamiana* leaves. Western blot analysis **(A)** and corresponding loading controls (RuBisCO large subunit) stained with Coomassie Brilliant Blue **(B)**, of pBet v 1a containing protein extract from leaves samples collected from 3 to 14 dpi. Each lane was loaded with 2.5 µg of TSP, the western blot was probed with anti-FLAG® antibody conjugated with horseradish peroxidase. Side numbers indicate molecular mass markers in kDa. p.c., positive control, 10 ng of a commercially available flagged protein; n.c., negative control, extract from leaves infiltrated solely with *A. tumefaciens* EHA105.

We first conducted a time-course expression analysis for the plants transformed with the vector pK7WG2.Betv1 to determine the day of maximum expression (**Figure 1**). A suspension of *A. tumefaciens* carrying the vector was manually infiltrated into the leaves. Three leaves were sampled daily, beginning 3 until 14 dpi. Western blot analysis of the leaf extracts revealed an expression peak at 3 dpi (**Figure 1**). The amount of pBet v 1a at the peak expression level was evaluated by densitometric analysis and corresponded to 5.1 ± 0.46 μg/g of fresh leaves weight (FLW).

#### pGPVXGATEWAY(A) Expression

**Figure 2:**
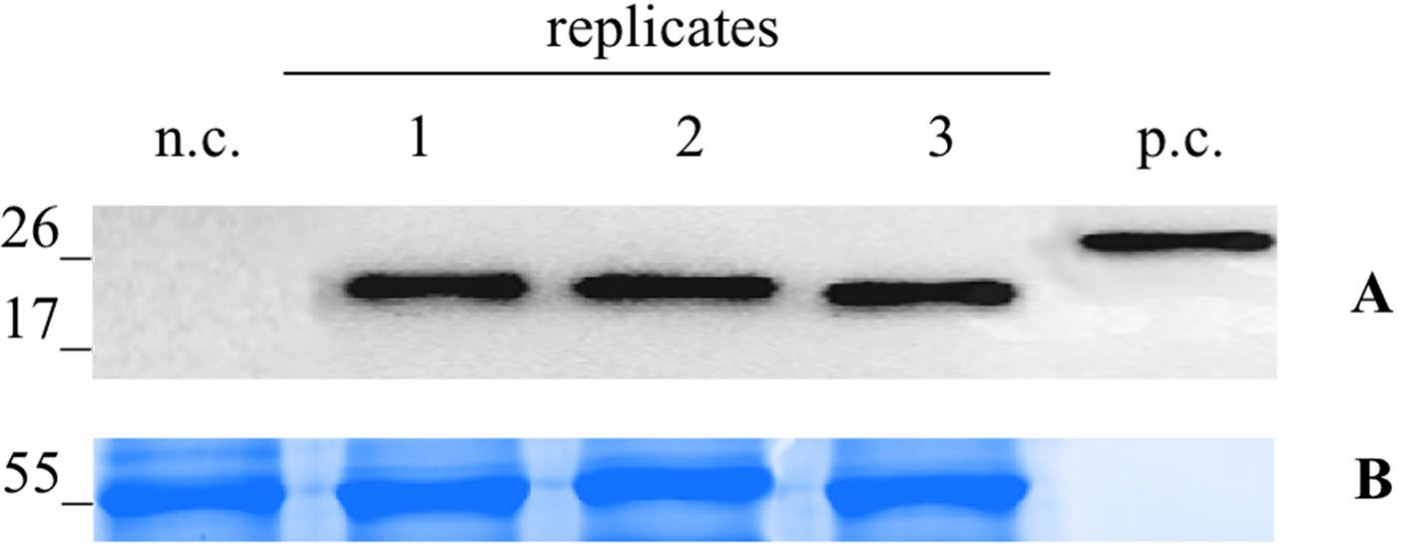
Evaluation of extraction reproducibilty of the pG PVX GATEWAY(A).Betv1 agro-infiltrated *N. benthamiana* leaves. Western blot analysis **(A)** and corresponding loading controls (RuBisCO large subunit) stained with Coomassie Brilliant Blue **(B)**, of pBet v 1a containing protein extract from leaves samples in three biological replicates. Each lane was loaded with 2.5 µg of TSP, the western blot was probed with anti-FLAG® antibody conjugated with horseradish peroxidase. Side numbers indicate molecular mass markers in kDa. p.c., positive control, 10 ng of acommercially available flagged protein; n.c., negative control, extract from leaves infiltrated solely with A. tumefaciens GV3101.

For the infection with the Potato Virus X (PVX)-based vector, the N. benthamiana leaves were infiltrated with the A. tumefaciens suspension carrying pGPVXGATEWAY(A).Betv1. When the first viral symptoms appeared (10 dpi), we collected the infected leaves and analyzed them by western blot analysis. In particular, three biological replicates consisting of a collection of the leaves of three infected plants (**Figure 2**) were performed. On the basis of the densitometric analysis, the expression level of the recombinant protein Bet v 1 was estimated to correspond to 51.9 ± 2.51 μg/g of FLW.

### pBet v 1a IMAC Purification and Yields

Small-scale purification experiments were set up, starting from plant material deriving from the two systems used for recombinant protein production in the *N. benthamiana* plants.

**Figure 3:**
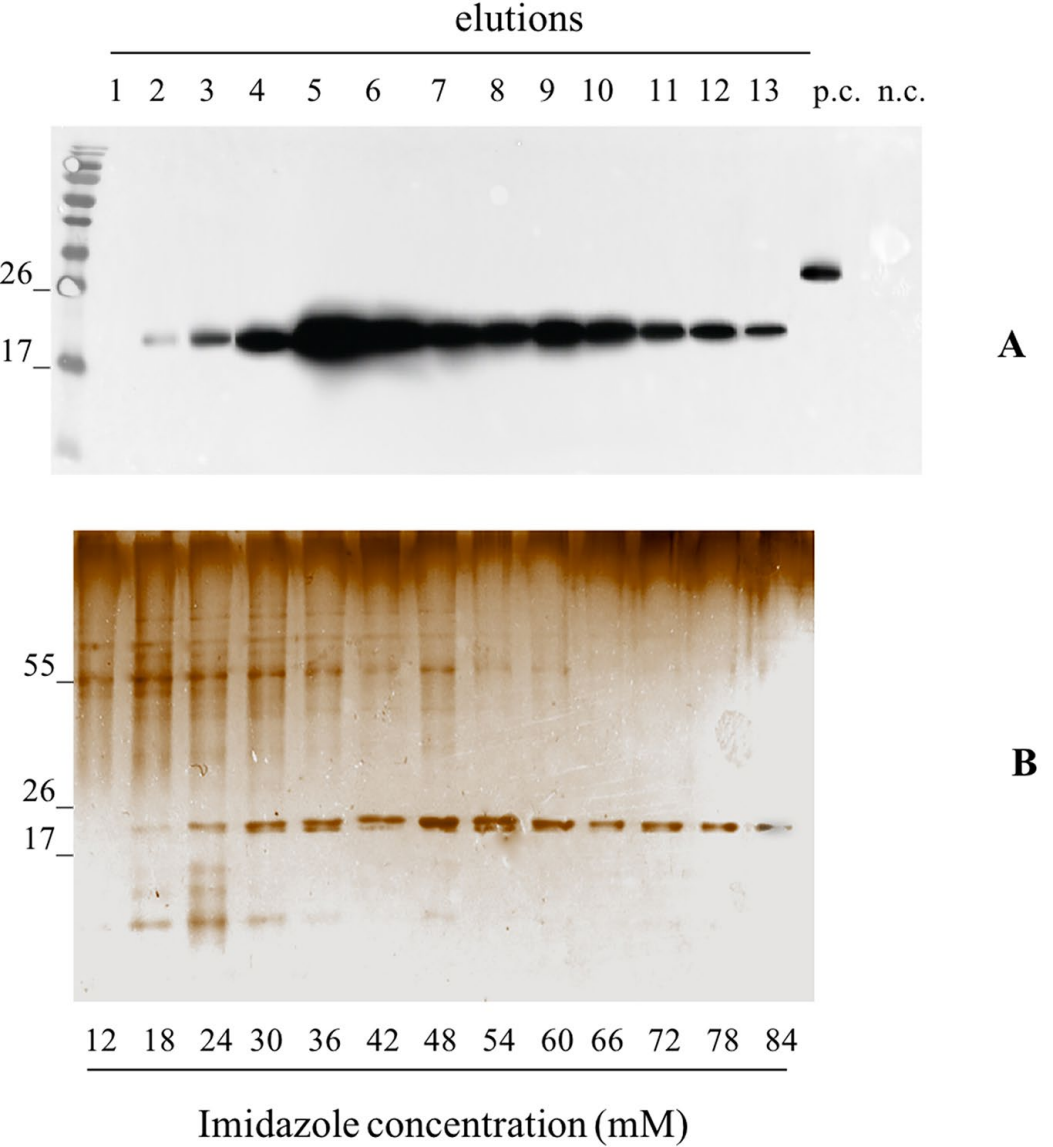
Evaluation of the pBet v 1a Purification, from pK7WG2. Betv1 agroinfected leaves, using a imidazole gradient. Western blot analysis **(A)** and corresponding Silver Staining **(B)**, of 1.5 mL purification elutions. Each lane was loadedwith 5 µL of elution, the western blot was probed with anti-FLAG® antibody conjugated with horseradish peroxidase. Side numbers indicate molecular mass markers in kDa. p.c., positive control, 10 ng of a commercially available flagged protein; n.c., negative control, from leaves infiltrated solely with *A. tumefaciens* EHA105, submitted to the same purification protocol as the leaves extract containing pBet v 1a. The bottom bar of the B image stands for the imidazole concentration for every gradient fraction.

We independently extracted the TSP starting from 30 g of leaves infiltrated with pK7WG2.Betv1 and 10 g of leaves infected using pGPVXGATEWAY(A).Betv1. The extracts were subjected to affinity chromatography using a Ni-NTA column (**Figure 3**). The optimal imidazole concentration for pure protein elution was between 55 and 60 mM (Fractions 10-13, **Figure 3**) and leaves infiltrated with pK7WG2.Betv1 and pGPVXGATEWAY(A).Betv1 displayed the same pattern (data not shown). In the discarded fractions 1 to 9, we detected a protein of approximately 55 kDa weight, which resulted to be a contaminant and not an oligomerization of pBet v 1a since it was not detected in the anti-FLAG® Western blot analysis. The yields of the purified recombinant protein obtained using the two systems were estimated by Western blot followed by a densitometric analysis of the protein bands recognized with the specific antibodies of three independent purified pBet v 1a preparations.

**Figure 4:**
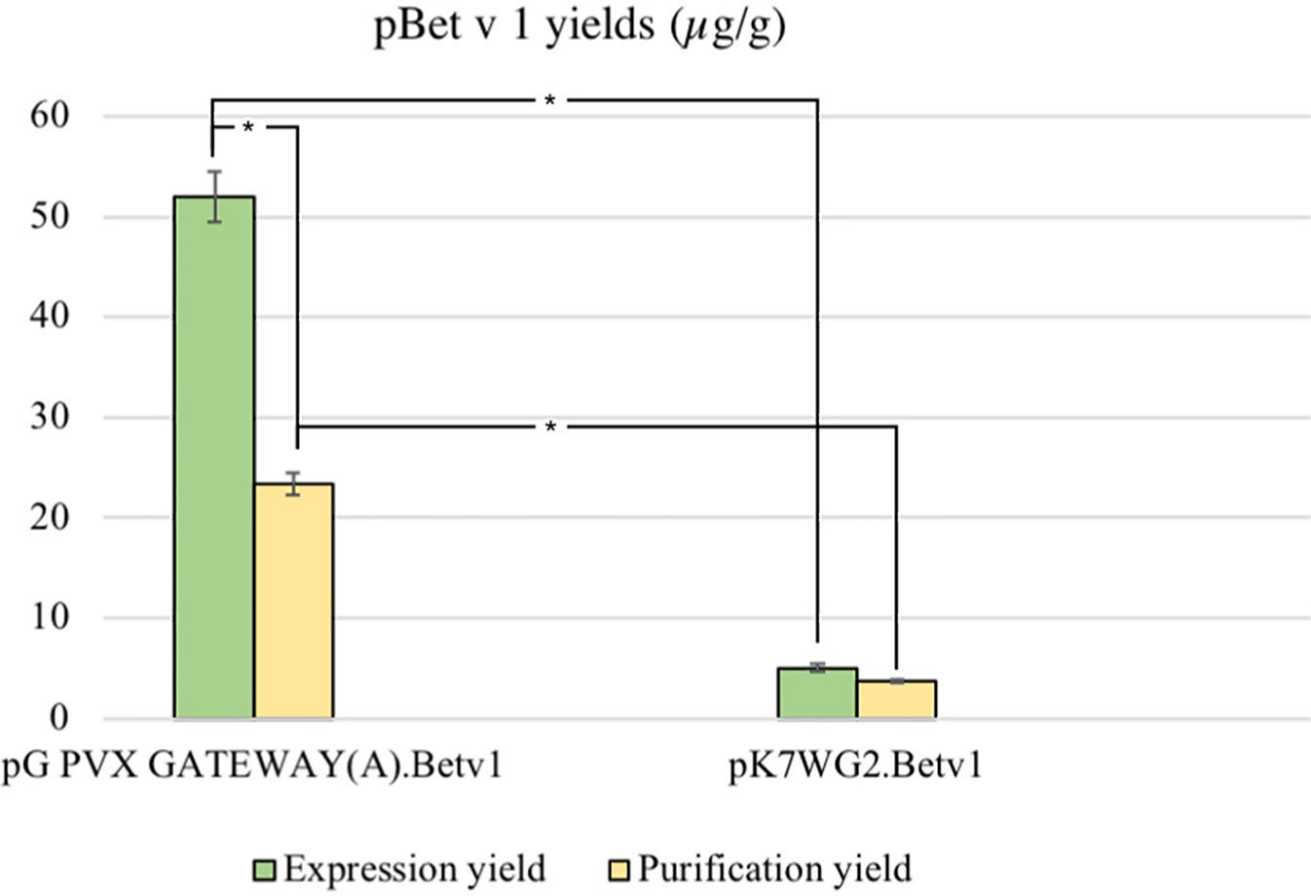
Average yields µg/g LFW of purified protein between the two expression systems. The expression and purification yields are reported, respectively, in green and yellow. The bars represent the standard deviations of three independent purification experiments, asterisks indicates the statiscal significance evaluated by t-test (*p* < 0.01).

The average yield of purified pBet v 1a obtained from pK7WG2.Betv1 and pGPVXGATEWAY(A).Betv1 was of 3.7 ± 0.02 μg/g FLW and 23.4 ± 0.004 μg/g FLW, respectively (**Figure 4**). The stardard deviation was calculated by comparing the results of three independent purification experiments for each expression system.

All the susequent experiments were performed using pBet v 1a produced using the pGPVXGATEWAY(A).Betv1 because of the higher yields obtained.

### Structural Characterization of pBet v 1a

#### Analysis of the pBet v 1a Preparation by Size Exclusion Chromatography and RP-HPLC

**Figure 5:**
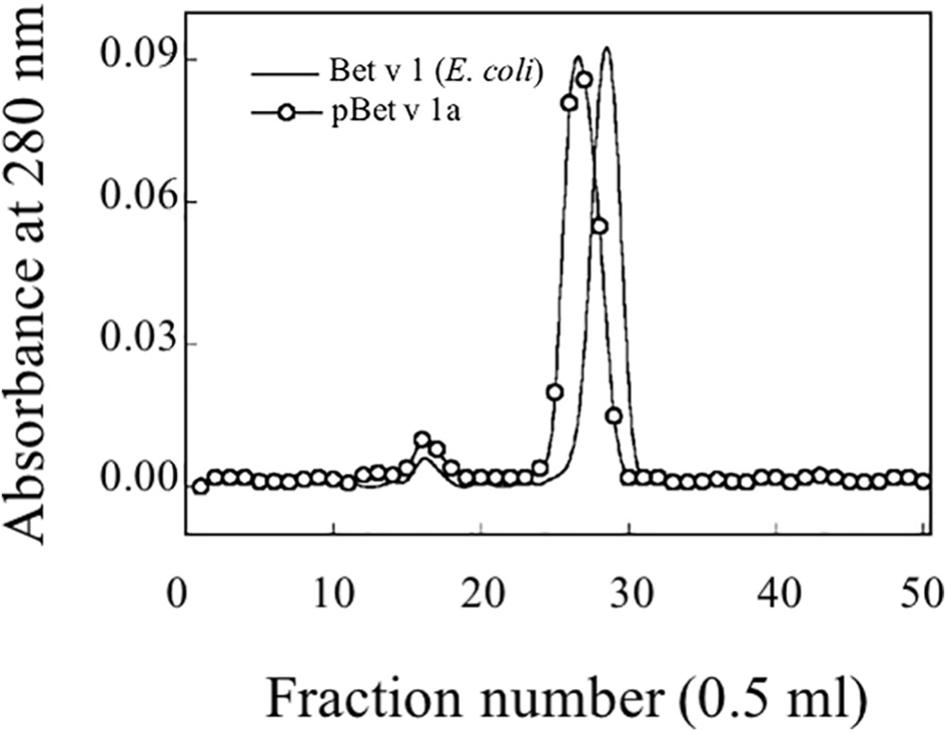
Gel filtration profile on a Superdex 75 column of purified pBet v 1a and Bet v 1 expressed in *E. coli*.

Following the affinity chromatography, pBet v 1a was subjected to size exclusion chromatography. **Figure 5** shows the elution profile of pBet v 1a compared with that of the allergen expressed in *E. coli*. Similar to the *E. coli* derived molecule, pBet v 1a was eluted as a single peak excluding the presence of aggregated forms. As expected, pBet v 1a is eluted at a slightly lower volume due to the higher molecular weight (19,696 Da) than that of the molecule expressed in E. coli (17,570 Da). This is due to the presence of a tail (containing a poly-Histidine tag, a Flag-tag and a linker) of 19 amino acid residues at the N terminus of pBet v 1a. The fractions containing pBet v 1a, eluted from the gel filtration, were collected and pooled. The concentration of the pure protein obtained from size exclusion chromatography was estimated on the basis of the molar extinction coefficient, at 280 nm.

**Figure 6:**
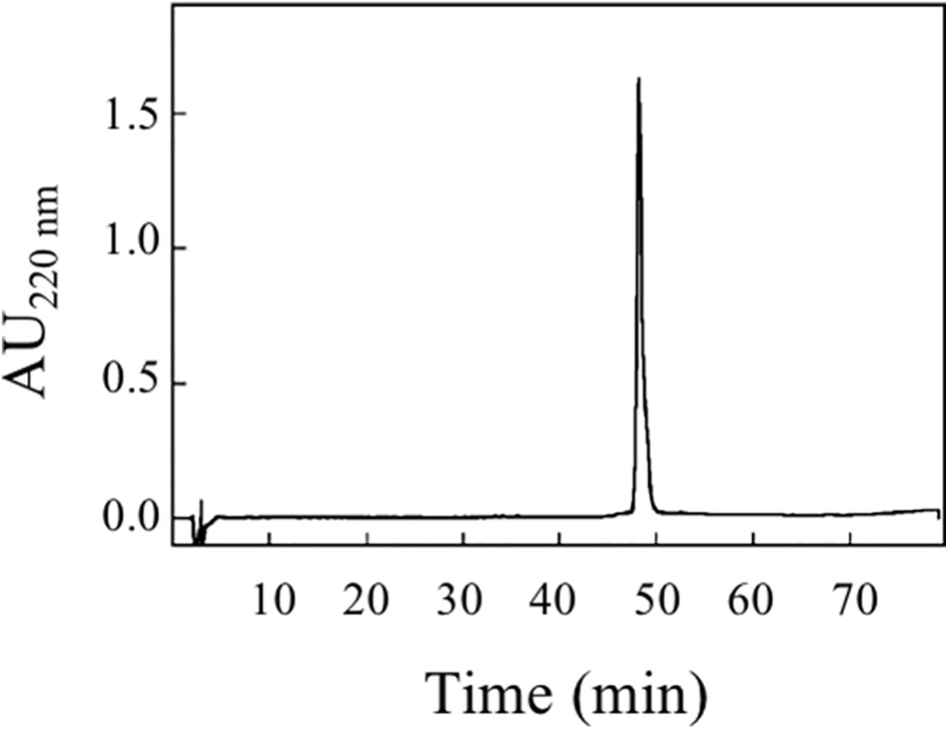
RP-HPLC elution profile of purified pBet v 1a (200 µg).

An amount of 0.2 mg was further analyzed by RP-HPLC (**Figure 6**). pBet v 1a was eluted as a single peak that was collected and concentrated with a centrifugal vacuum concentrator (Savant Speedvac Plus SC110A).

#### Assessment of pBet v 1a Identity by Mass Spectrometry

**Figure 7:**
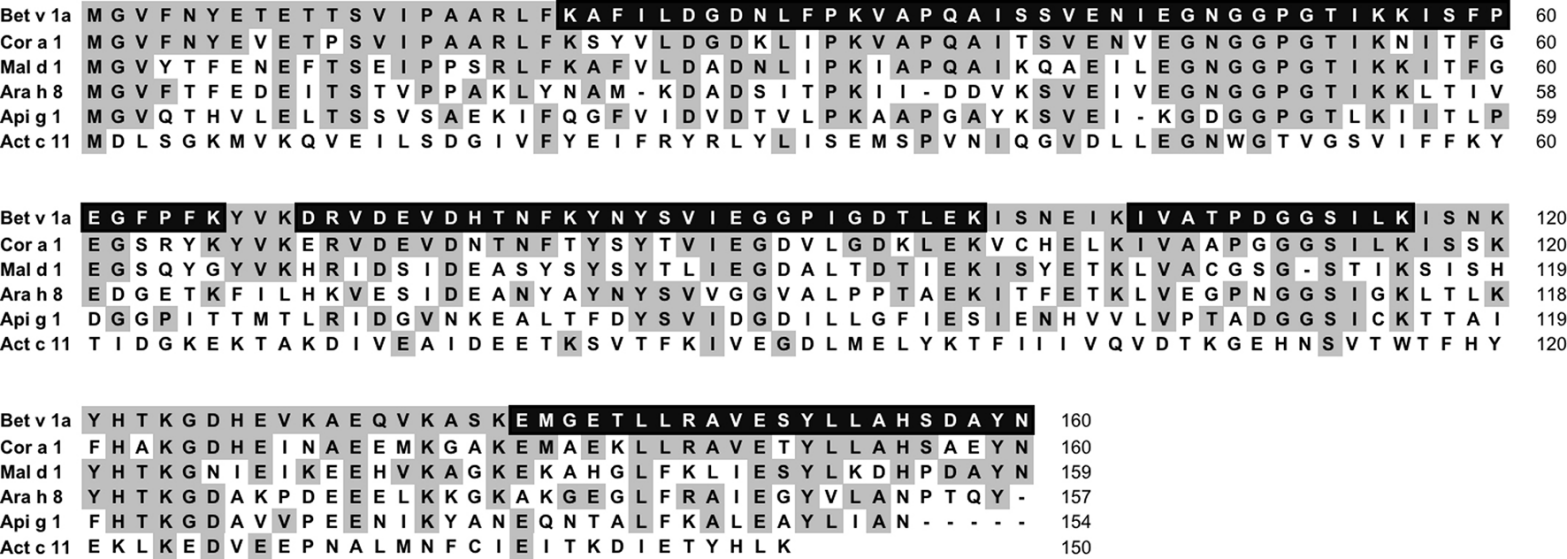
Multiple-sequence alignment of Bet v 1a (accession number P15494) with the homologous allergens Cor a 1, Mal d 1, Ara h 8, Api g 1 and Act c 11 having the accession number Q08407, 598 Q9SYW3, Q6VT83, P49372, A0A2R6PAW0, respectively. The residues of Bet v 1a conserved in the aligned homologues are shadowed in gray. The amino acid residues experimentally identified by mass spectrometry are in white and black shadows.

An amount of Bet v 1a corresponding to 5 µg, deriving from RP-HPLC elution, was used for the assessment of its identity using the in solution enzyme digestion method followed by shotgun proteomics (Shan et al., 2013). This procedure allowed the identification of several overlapping peptides covering 76% of the Bet v 1a protein sequence (see **Figure 7**). Except a peptide deriving from the trypsin enzyme used for the protein digestion, peptides belonging to any other protein contained in Uniprot database were not detected. In addition, no sequence heterogeneities were observed. Therefore, this experiment demonstrates that the plant expressed pBet v 1a was exactly the expected protein and that the molecule had been purified to homogeneity.

In summary, the purity of pBet v 1a eluted from size exclusion chromatography was assessed using different methods. It provided a single band on SDS-PAGE, was eluted as a single peak from RP-HPLC and was identified as a single molecule from shotgun proteomics. No evidence of the presence of post-translational modifications was recorded.

#### Structural Analysis by Circular Dichroism Experiments

**Figure 8:**
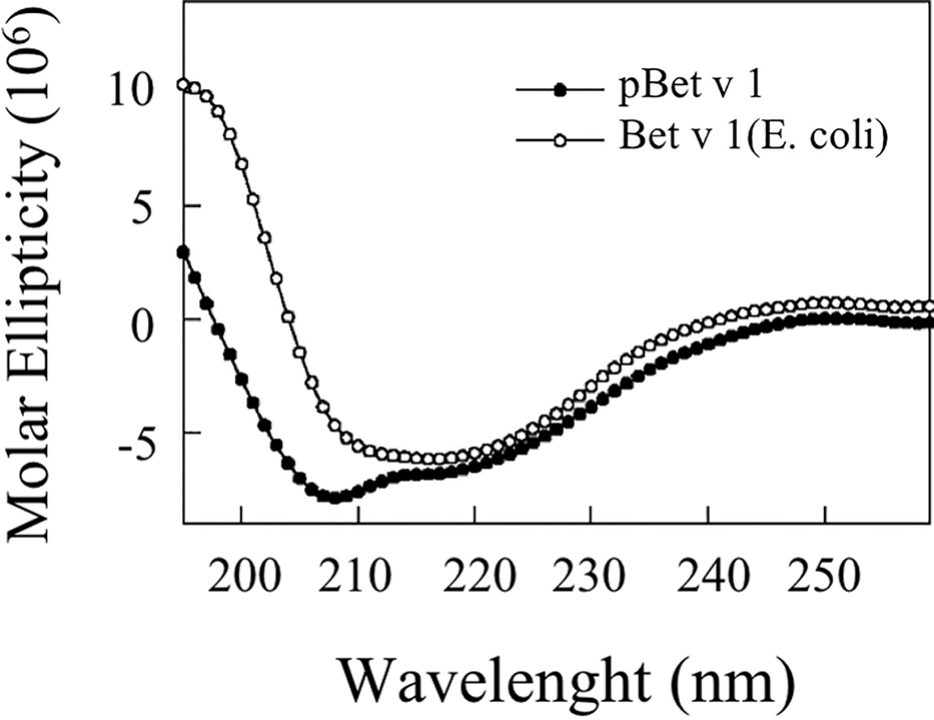
CD spectra of plant-expressed and *E. coli*–expressed (commercial) Bet v 1.

**Figure 8** shows that the CD spectrum obtained for pBet v 1a is different from that of the *E. coli*–produced allergen. The CD curve of the bacterial-derived allergen is very similar to the one reported in literature for the natural Bet v 1 (Batard et al., 2005; Bollen et al., 2007), displaying a broad minimum around 218 nm. Conversely, the spectrum of the pBet v 1a shows two minima around 208 and 216 nm, consistent with a ratio of the alfa-helix higher than that present in the structure of the molecule expressed in *E. coli*.

#### Thermal Stability

**Figure 9:**
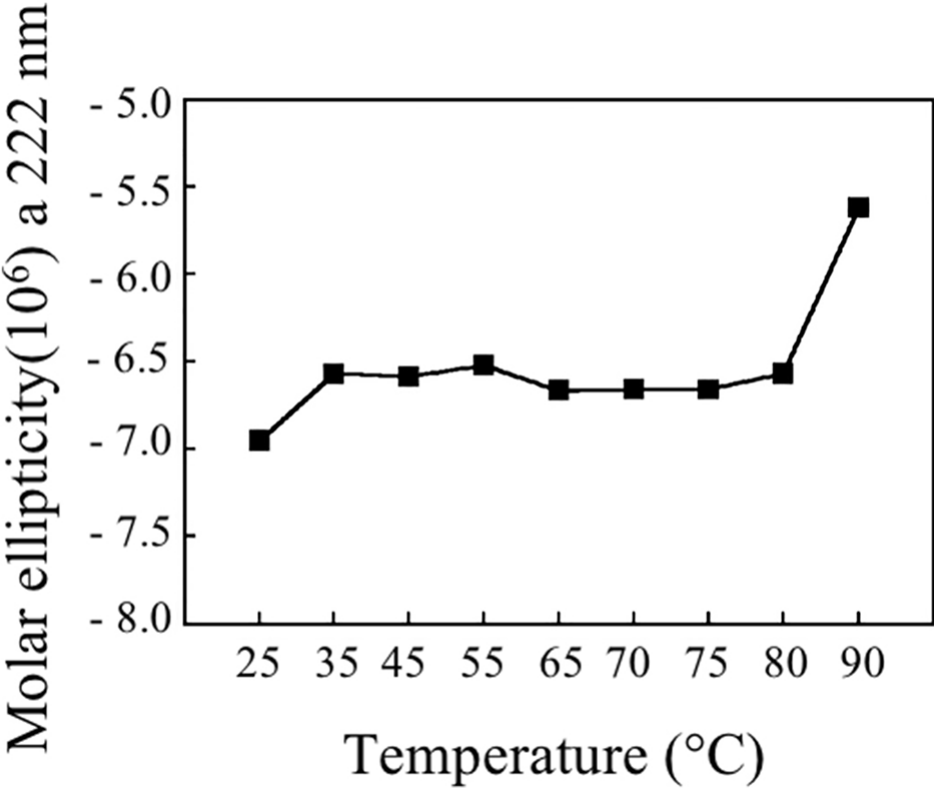
Molar ellipticity of pBet v 1a at 222 nm at the analyzed temperatures.

**Figure 9** shows the stability of Bet v 1a at different temperatures, reported as molar ellipticity registered at 222 nm. It can be observed that the protein maintains its structure until 80°C and only at 90°C the unfolding starts.

### Immunological Characterization

#### IgE-Binding Inhibition

**Figure 10:**
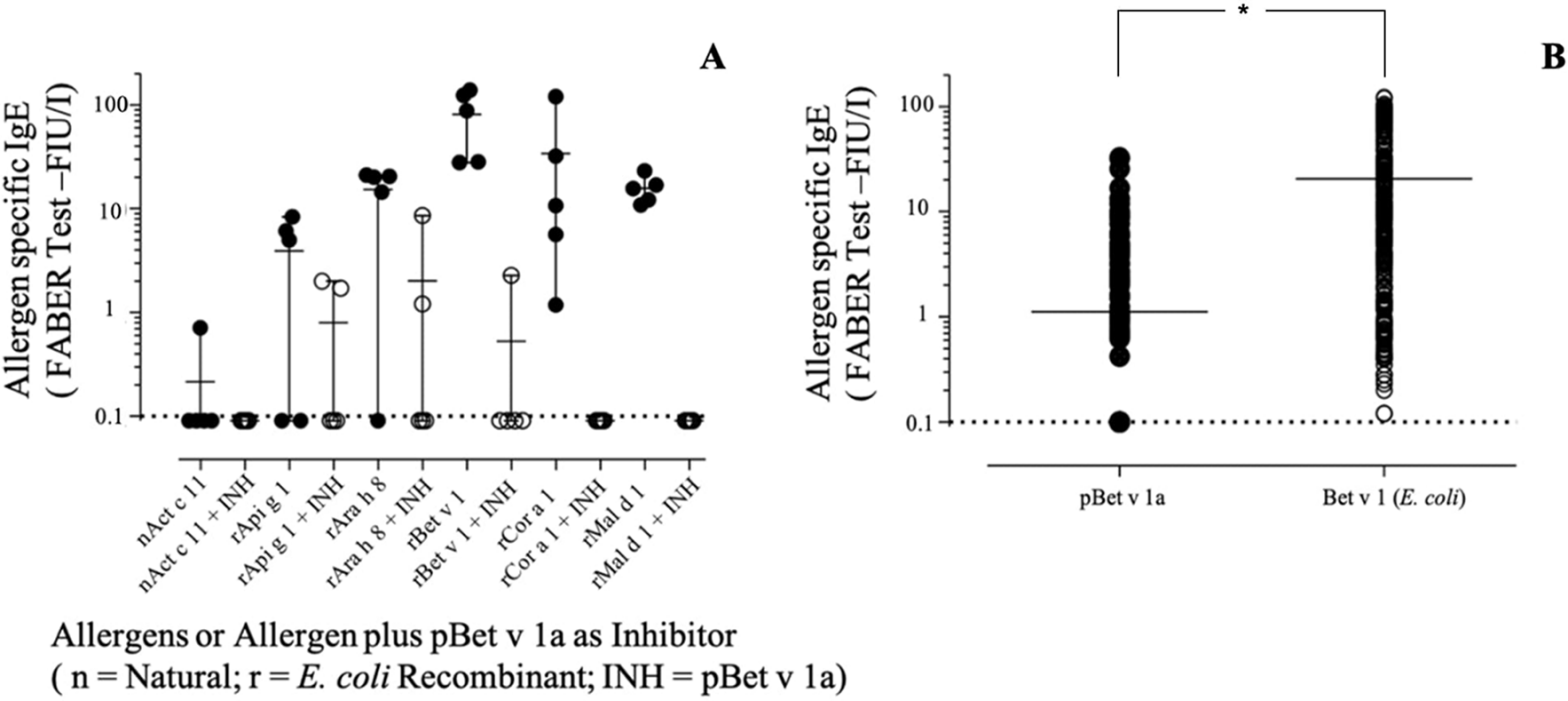
**(A)** Specific IgE-binding inhibition using pBet v 1a on Bet v 1-like molecules on the FABER test solid phase. **(B)** Specific IgE direct comparative binding using 304 Bet v 1 IgE-positive sera and statistical evaluation of IgE value distributions by using the t-test for paired values (*p* < 0.0001), displayed by the asterisk.

As shown in **Figure 10A**, pBet v 1a used in solution to inhibit the IgE binding to the *E.coli*–produced Bet v 1 was able to provide an inhibition of up to 100%. The same was obtained for Cor a 1 and Mal d 1, whereas an almost complete inhibition was achieved with two other Bet v 1-like molecules from peanut and celery. These results are in line with the different degree of primary structure similarity observed by comparing the sequence of Bet v 1a with those of the homologous allergens analyzed in this study (**Figure 7**). In fact, the sequence identity between Bet v 1a and the Bet v 1-like allergens Cor a 1, Mal d 1, Ara h 8 and Api g 1 is 72.5%, 55.6%, 46.2% and 40.0%, respectively. Therefore, a clear correlation between the level of IgE binding inhibition and the primary structure similarities is observed. IgE binding inhibition to kirola, Act c 11, was also observed, although the sequence identity with Bet v 1a is very low (12%). However, Act c 11 (as well as Act d 11) represents a particular case since it is not included in the Bet v 1-like protein family. In fact, it belongs to the Major Latex Protein/Ripening Related Protein (MLP/RRP) family, but it is immunologically correlated with Bet v 1-like allergens with which it displays IgE co-recognition (D’Avino et al., 2011; Chruszcz et al., 2013).

#### Direct IgE Binding

When the pBet v 1a was tested for direct comparison with the one produced in *E. coli* on an extended number of samples, the values were differently dispersed with a mean IgE value lower for pBet v 1a (**Figure 10B**). The observation of single 304 IgE values showed that some sera had almost overlapping reactivities, whereas others showed a markedly different behavior, recording a number of negative results. Comparing the IgE value distribution by using the t-test for paired values, it turned out that the series were statistically different (*p* < 0.0001).

The best performing sera belonged to the Bet v 1 IgE-positive subset having IgE reactivities also on other Bet v 1-like molecules (data not shown).

## Discussion

The development of tools for a precise and definitive diagnosis of allergic diseases is considered strategic for the future, both in terms of developing vaccines for immunotherapy, exploiting a companion diagnostic strategy, and of studying the genuine allergic sesitization of patients to a particular allergen source. Determining the sensitization profile of an inidvidual patient creates the opportunity to assess the individual risk of the severity of an allergic reaction and to predict the natural course (Van Gasse et al., 2015).

The progress in molecular farming exploiting different production platforms together with the current knowledge of allergen components and protein families (www.allergen.org; www.allergome.org) has boosted molecular allergy diagnosis both for food and for inhalant allergens. In this context, plant-based expression systems are considered advantageous for several reasons: (1) cost-effectiveness, (2) ease in scalability, and (3) authenticity with respect to plant-derived allergens.

**Figure 11:**
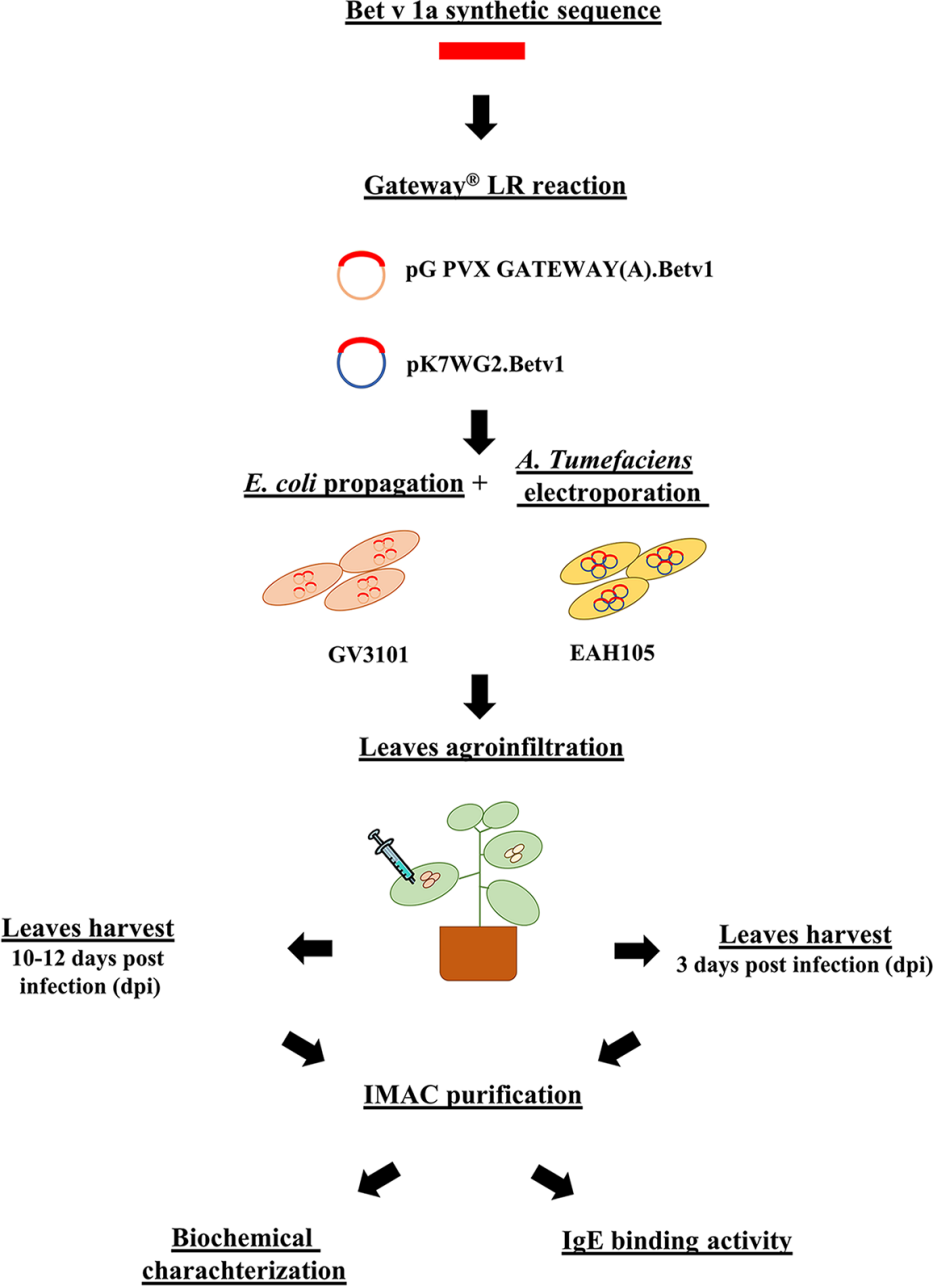
Streamline pBet v 1a production and characterization.

In this study, we have investigated and compared the performance of two different strategies for the transient expression of allergenic proteins in plants, one based on the use of a modified plant-virus and the other on a plant expression vector. The pipeline of the two different strategies is visualized in **Figure 11**. The virus-based mediated a higher expression level, almost ten-fold, than the plant one, as previously demonstrated for other recombinant proteins (Salazar-González et al., 2014). This result may be explained in terms of the higher rate of transcription of viral RNA-dependent RNA polymerase and of the natural capacity of viruses to sequester the plant apparatus for their replication in the host. However, the highest expression levels here obtained were four times (Krebitz et al., 2000) lower than those previously reported in *N. benthamiana* using a tobacco mosaic virus (TMV)-based system for the expression of the same protein. We speculate that this difference may be explained either in terms of a lower efficiency of PVX than TMV in mediating foreign protein expression or of the presence in our construct of a His-tag which may influence the recombinant protein accumulation (Pinnola et al., 2015), or even as a result of a combination of the two factors.

pBet v 1a was purified for the first time to our knowledge from *N. benthamiana* infected and infiltrated leaves using a single-step purification protocol based on affinity chromatography. The recombinant protein was purified at higher absolute yields when using the viral vector in comparison to the use of the plant-specific expression vector. However, the former was characterized by lower relative yields, probably reflecting a lower efficiency of the affinity column in capturing the antigen, this being present in higher amounts in the extracted plant soup.

Bet v 1 is the major allergen of birch pollen. The expression of this protein was chosen for this project because it is an important and well-studied allergen for which reference information is available in the literature. In addition, the recombinant molecule from a prokaryotic expression system is available for comparative analysis. In particular, *E.coli*–made Bet v 1 is already used in *in vitro* allergy diagnosis, and it is available as an allergen reference standard from the European Directorate for the Quality of Medicines and Health Care, EDQM (Vieths et al., 2012).

The pBet v 1a produced in this study was recognized by the specific IgE of patients allergic to birch pollen and positive to *E. coli*–made Bet v 1. It efficiently bound specific IgE in solution, thus inhibiting their binding with Bet v 1 and its homologs that were immobilized on the FABER biochip. Nevertheless, in some experimental conditions, pBet v 1a showed a different behavior with respect to the molecule expressed in the prokaryotic system. For instance, lower IgE values were often obtained for pBet v 1a when it was spotted on the FABER biochip and tested simultaneously with *E. coli*–made Bet v 1. The results, obtained from an investigation of the structural features of pBet v 1a, suggest that its individual immunological features might be associated with a molecule conformation different from that of the prokaryotic Bet v 1 used for comparison. In fact, CD experiments showed a higher content of the helical structure in pBet v 1a than in the *E. coli*–made protein. This is not very surprising since it is well known that the Bet v 1 conformation is affected by various factors. Its structure is not stabilized with disulphide bridges (Radauer et al., 2008), as occurs for allergens that have a very compact structure, such as LTP and gibberellin-regulated proteins (Tuppo et al., 2014; Giangrieco et al., 2015). Although we cannot exclude the possibility that the presence of the histidine tail and flag, added at the N-terminus of pBet v 1a, could affect its conformation. An additional factor worth considering is the Bet v 1 ligand binding (Śliwiak et al., 2016). In fact, Bet v 1 and its homologs are described as a promiscuous acceptor for a wide variety of hydrophobic ligands (Chruszcz et al., 2013; von Loetzen et al., 2013). In particular, the ligand deoxycholate has been reported to stabilize the protein conformational IgE epitopes. It has also been suggested that ligand-binding affects the allergenicity of the protein and that humans are exposed to both ligand-bound and ligand-free Bet v 1 (Asam et al., 2014). Therefore, the absence or possible presence of a specific ligand bound to pBet v 1a and affecting its structure and immunological behavior is something worth investigation in the near future. Sometimes, as reported also for the kiwifruit allergen Act d 5 (Bernardi et al., 2010), the availability of an allergen with different conformations is useful to have a greater number of panels of the IgE epitopes, which can be used to reveal additional sets of IgE antibodies, thus improving allergy diagnosis.

Thermostability experiments suggest that pBet v 1a might be more stable than the molecule expressed in *E. coli* and lacking the 19-residue tail present at the N-terminus of the molecule described in this study. In fact, pBet v 1a is stable until 80°C and only at 90°C starts to loose its secondary structure elements. In contrast, a melting point of 66°C was reported for Bet v 1 expressed in *E. coli* (Himly et al., 2009). Further studies are necessary to understand whether the 19-residue tail of pBet v 1a has a stabilizing effect on the structure of this molecule.

In conclusion, the results of this study highlight the valuable performance of the plant-based expression system tested with the major birch allergen Bet v 1a. This represents a feasible platform for the production of the major allergen of birch, being characterized by high yields and a simple, one-step, protocol for recombinant protein purification. We, therefore, suggest that this platform may be used succesfully also for other recombinant allergens whose production is not feasible in bacterial cells. In addition, this technique could be exploited for the development of immunotherapeutic strategies, also considering their potential for oral administered vaccines. In fact, as recently demonstrated for other allergens (Fukuda et al., 2018), the administration to humans of molecules deriving from edible plant organs can be a safer approach than using those obtained from non-edible sources.

## Author Contributions

MS performed the transient pBet v 1a expression in plants, its characterization and purification. IG performed the structural characterization of the expressed protein by CD, size exclusion chromatography, RP-HPLC and thermal stability experiments and conjugated pBet v 1a to the nanobeads for the FABER test. MAC contributed in manuscript writing. RZ supervised the upstream and downstream process set-up. MP contributed in the experimental design. CR, MC, and AM designed, performed, and supervised all the immunological experiments. LA designed the experiments, coordinated all the experimental activities, and wrote the manuscript.

## Conflict of Interest

The authors declare that the research was conducted in the absence of any commercial or financial relationships that could be construed as a potential conflict of interest.
